# Monitoring the Corrosion Process of Reinforced Concrete Using BOTDA and FBG Sensors

**DOI:** 10.3390/s150408866

**Published:** 2015-04-15

**Authors:** Jianghong Mao, Jiayun Chen, Lei Cui, Weiliang Jin, Chen Xu, Yong He

**Affiliations:** 1 Ningbo Institute of Technology, Zhejiang University, Ningbo 315100, China; E-Mails: jhmao@nit.zju.edu.cn (J.M.); jinwl@zju.edu.cn (W.J.); 2 Institute of Structural Engineering, Zhejiang University, Hangzhou 310058, China; E-Mails: chanjiayun@gmail.com (J.C.); zju_xuchen@zju.edu.cn (C.X.); yong.he1979@gmail.com (Y.H.)

**Keywords:** distributed optical fiber sensor, fiber Bragg grating sensor, structural health monitoring, corrosion, expansion monitoring, cracking detection

## Abstract

Expansion and cracking induced by the corrosion of reinforcement concrete is the major factor in the failure of concrete durability. Therefore, monitoring of concrete cracking is critical for evaluating the safety of concrete structures. In this paper, we introduce a novel monitoring method combining Brillouin optical time domain analysis (BOTDA) and fiber Bragg grating (FBG), based on mechanical principles of concrete expansion cracking. BOTDA monitors concrete expansion and crack width, while FBG identifies the time and position of cracking. A water-pressure loading simulation test was carried out to determine the relationship between fiber strain, concrete expansion and crack width. An electrical accelerated corrosion test was also conducted to evaluate the ability of this novel sensor to monitor concrete cracking under practical conditions.

## 1. Introduction

Reinforcement corrosion has become the major reason for the failure of concrete durability. It is estimated that maintenance costs for reinforced concrete structures is up to 100 billion dollars worldwide [1]. The occurrence and development of reinforcement corrosion has adverse effects on both service and safety aspects of concrete performance. For example, when bridges are exposed to chloride or to deicing salt, reinforcement corrosion leads to the expansion and destruction of the concrete cover, exposing the reinforcement to the corrosive environment and resulting in accelerated corrosion of the steel. Therefore, it is necessary to monitor the progression of reinforcement corrosion in order to guarantee the safety of the reinforced concrete structures.

Newly developed technologies, such as the anode ladder sensor developed by RWTH Aachen University [2] and the electrode array sensor developed by Queen’s University of Belfast [3], facilitate the monitoring of reinforcement corrosion. These sensing technologies, which operate by measuring the electrochemical characteristics of steels, can indicate the occurrence of reinforcement corrosion, but cannot assess the degree of corrosion. Based on the mechanical model of concrete expansion cracking [4], concrete strain increases as corrosion products fill the voids during the progression of reinforcement corrosion, which results in cracking of the concrete cover. Traditional sensing technologies (e.g., resistance strain gauges and vibrating wire strain gauges) cannot monitor corrosion, due to a number of unfavorable properties, such as large volume, electromagnetic interference and poor corrosion resistance. In contrast, optical fiber sensing technology is remarkable for its implantability, anti-electromagnetic interference and durability, which includes chloride concentration detection [5] and pH measurement [6]. However, these sensors are only able to detect parameters related to the corrosion environment, but not the strain induced by reinforcement corrosion.

In recent years, the fiber Bragg grating (FBG) sensor has been extensively adopted as a new non-destructive evaluation technique for monitoring strain in structures under corrosive conditions [7]. Saouma [8] and Holton [9] bonded FBG sensors onto the steel bar of cast-in-place concrete structures in order to monitor the strain response *in situ*. Lee [10] used FBG sensors to measure steel strain during the process of corrosion. Hassan [11] presented an 80% etched-cladding fiber Bragg grating sensor to monitor the production of corrosion waste in a localized region of the rebar. Distributed optical fiber sensors, named Brillouin optical time domain analysis (BOTDA) or Brillouin optical time domain reflectometer (BOTDR), are another novel optical monitoring technology developed in the last decade. They can achieve synchronous strain monitoring of structures both in multi-environment and multi-dimensional space [12]. Zhao [13] demonstrated that BOTDA is feasible for realizing durability monitoring, in which a sensing fiber is twined around the steel rebar. Sun [14] approved that BOTDR can accurately measure the strain values and identify the crack locations of the simulated reinforced concrete column. The authors of this paper [15,16] also carried out experimental research on reinforced concrete corrosion monitoring based on BOTDA, and the results showed that BOTDA can record expansive deformation and estimate the cracking width. However, due to the limitation on spatial resolution, it is impossible to identify the initial cracking moment and position of cracks.

In this paper, we present an approach that combines BOTDA and FBG. BOTDA is used to monitor the progression of expansion and cracking induced by reinforcement corrosion, while FBG is used to identify the time and position of cracking. A water-pressure loading simulation test was used to establish a relationship between fiber strain, concrete expansion and crack width. An electrical accelerated corrosion test was also carried out to verify the effectiveness of the monitoring of concrete cracking under practical conditions.

## 2. Materials and Methods

### 2.1. Principle of BOTDA and FBG Sensors

#### 2.1.1. Principle of BOTDA Sensors

The interaction between incident light waves and acoustic phonons in optical fibers generates Brillouin scattered light as backscattered light, which propagates in the opposite direction of the incident light waves. When the strain or temperature varies, the Brillouin frequency will shift. The frequency shift has a linear relationship with both strain and temperature. The relationship among strain variation, temperature change and Brillouin frequency shift is expressed in Equation (1) [17].

(1) $$\Delta v_{\text{B}}(\varepsilon ,T)=\frac{dv_{\text{B}}(T)}{dT}\Delta T+\frac{dv_{\text{B}}(\varepsilon )}{d\varepsilon }\Delta \varepsilon$$

The Brillouin frequency shift Δv_B_(*ε*,*T*) has a relationship with both temperature change (Δ*T*) and strain variation (Δε), but there is no coupling relationship between them. Free optical fiber sensors that are not bonded to a structure can be used as temperature compensation sensors by laying them beside the strain monitoring sensors, thus eliminating the influence of temperature on strain monitoring.

*Z* is the distance from the position where incident light is launched to the position where scattered light is generated, which can be determined by using Equation (2).

(2) $$Z=\frac{\text{c}\cdot t}{2n}$$

where *c* is the velocity of light in a vacuum, *n* is the refractive index of the optical fiber and *t* is the time interval between launching the incident light and receiving the scattered light at the launching end.

As a distributed testing technique, BOTDA’s main disadvantage is its limited spatial resolution. Spatial resolution is determined by the pulse width (*τ*) of the incident light. For a given pulse width *τ*, the spatial resolution ∆Z is expressed as:

(3) ΔZ=ντ/2

where ν represents the light velocity in the optical fiber. As a result, the strain of every sample point is determined by averaging strains of all sample points located inside the spatial resolution section [18].

#### 2.1.2. Principle of FBG Sensors

FBG is a periodic and permanent modification of the core refractive index value along the optical fiber axis [19]. The FBG sensor operates by measuring the changes in the reflective signal from the grating, which is influenced by the external parameters of the surrounding material. The Bragg wavelength (*λ_B_*) that is reflected at the sensor is given by:

(4) $$\lambda_{B}=2n_{B}\Lambda$$

where *n_B_* is the reflective index and Λ is the spatial pitch. When projecting a wide spectrum light source onto the optical fibers, the narrow band spectrum satisfying Equation (4) would be reflected back due to the effect of the gratings.

The relationship between the reflective Bragg wavelength shift (Δ*λ_B_*) and the changes of strain and temperature at the grating region (ε*_g_*) can be expressed as:

(5) $$\frac{\Delta \lambda_{B}}{\lambda_{B}}=(1-\rho_{\varepsilon })\Delta \varepsilon_{g}+\left(\alpha +\beta \right)\Delta T$$

where *ρ_ε_* is the strain-optic coefficient, ν is Poisson's ratio and α and β represent the thermal expansion and thermo-optic coefficients, respectively. Any changes in external parameters (e.g., temperature, pressure, *etc*.) would alter the grating characteristics and result in a shift of the reflected Bragg wavelength.

### 2.2. Sensor Design for Monitoring the Corrosion Process of Reinforced Concrete

Once the chloride concentration around steel exceeds a threshold value, the passive film is disrupted or destroyed, resulting in spontaneous corrosion of the steel. The volume of rust products is about four- to six-times larger than that of iron. This growth of volume induces internal tensile stress in the cover concrete, and when the stress exceeds the tensile strength of the concrete, the concrete cover will be damaged by cracking.

A three-stage theory has been generally accepted as a reasonable description for reinforced concrete corrosion: (1) free expansion stage: the corrosion products fill the voids around the steel/concrete interface or migrate away from the steel/concrete interface; (2) stress-initiated stage: as the total amount of corrosion products reaches the threshold value, the formation of corrosion products starts to create expansive stress on the surrounding concrete, which increases with the growth of corrosion products; (3) cracking stage: when the expansive stress exceeds the tensile strength of the concrete, cracks will initially emerge around the steel and subsequently propagate to the surface of the concrete [20]. In this paper, we use this understanding of the mechanism of concrete corrosion to design a novel corrosion sensor based on BOTDA and FBG.

#### 2.2.1. Monitoring of Corrosion-Induced Concrete Expansion by BOTDA

Concrete is under elastic deformation before cracking; therefore, elastic mechanics principles are used to explain the mechanism of corrosion-induced concrete expansion. In this paper, three main assumptions are considered:

(1) Elastic deformation: concrete behaves as an elastic material, such that non-linear deformation is not considered.
(2) Uniform corrosion: corrosion products arrange uniformly around the steel; therefore, the expansion stress acting on the concrete cover is uniform.
(3) No leakage of corrosion products: corrosion products do not leak out from the concrete cover; therefore, expansion stress increases with increasing amounts of corrosion products.

**Figure 1:**
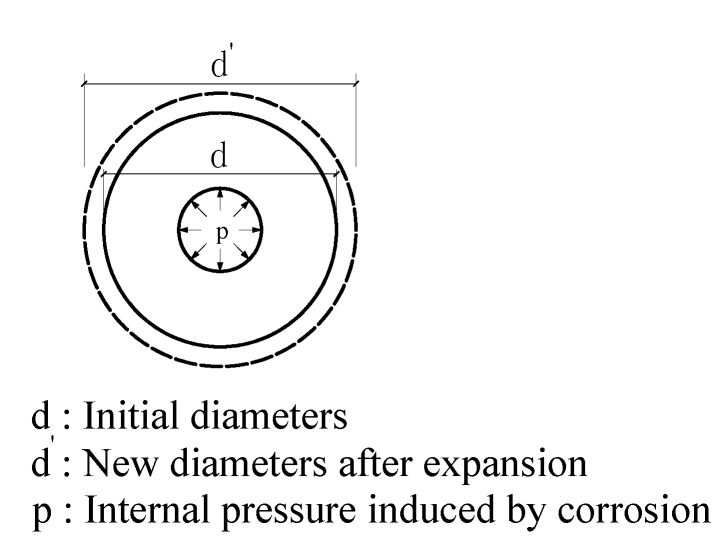
Mechanical model of reinforced concrete corrosion.

The mechanical model of reinforced concrete corrosion is illustrated in Figure 1:

According to elastic mechanics theory, the mechanical equation for a circular ring bearing uniform internal stress is described in Equations (6)–(8).

(6) $$\sigma =\frac{a^{2}p}{b^{2}-a^{2}}(\frac{b^{2}}{r^{2}}+1)$$

(7) $$\pi d^{'}=\pi d(1+\varepsilon )$$

(8) $$\Delta d=d\varepsilon =d\frac{\sigma }{E}=\frac{a^{2}pd}{(b^{2}-a^{2})E}(\frac{b^{2}}{r^{2}}+1)$$

where *σ* is the hoop stress of concrete, *p* is the internal pressure, *b* is the external diameter of the circular ring, *a* is the internal diameter of the circular ring, *r* is the distance from the analysis point to the center, ε is the hoop strain at the analysis point and *E* is the elastic modulus of concrete. From the equations above, it is clear that there is a linear relationship between corrosion-induced expansion (Δ*d*) and concrete strain (*ε*).

Therefore, one can estimate concrete expansion by monitoring hoop strain. In this paper, optical fibers were bent into a circular shape around a steel bar to record concrete expansion strain by BOTDA.

#### 2.2.2. Identifying Corrosion-Induced Concrete Cracks by FBG

As discussed in Section 2.1, BOTDA has limited spatial resolution. Thus, it is impossible for BOTDA to determine the position of cracking. However, according to the basic theory of concrete behavior, after a concrete material develops a crack, the concrete strain around the crack is released, resulting in decreased concrete strain next to the crack. A bare FBG sensor is characteristically flexible and precise, rendering it suitable for identifying corrosion-induced cracking. In this paper, several bare Bragg grating gauges were pasted on the surface of circular specimens alongside a distributed optical fiber, and the variations of wavelength were recorded by FBG.

#### 2.2.3. Monitoring the Width of Corrosion-Induced Concrete Cracks by BOTDA

**Figure 2:**
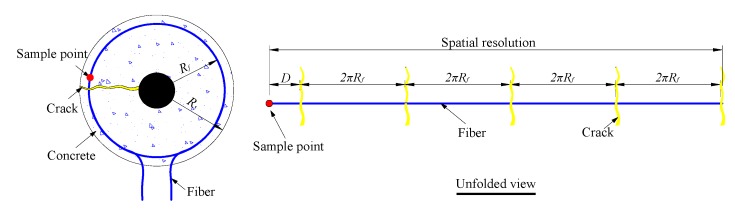
Illustration for crack detection by Brillouin optical time domain analysis (BOTDA).

Elastic mechanics theory is inapplicable once concrete cracks have formed. Thus, there is no mechanical theory that explains the relationship between crack width and optical fiber strain. However, geometric analysis can be utilized to establish the relationship between crack width and optical fiber strain. The strain at positions where the optical fiber crosses a crack is greater than the strain at other positions. Therefore, crack width analysis can ignore the strain measured at all points other than the crack position. According to geometric analysis, the strain at positions where the optical fiber crosses a crack is given by:

(9) $$\varepsilon =\frac{\Delta L}{L}=\frac{w}{\Delta z}$$

where *w* is the total widths of all cracks, ε is the optical fiber strain, *ΔL* is the elongation length of the optical fiber due to cracking (equal to total crack widths *w*) and *L* is the gauge length (*L* is equal to the spatial resolution of BOTDA). The illustration of crack detecting by BOTDA is shown as Figure 2.

In Figure 2, according to the spatial resolution of BOTDA discussed in Section 2.1.1, the strain of each sample point is affected by several cracks. The verification test has been done to prove that the spatial resolution was equal to the length of the fiber, at the head of which the sample point is located [12]. Learned from the unfolded view in Figure 2, the number of cracks (*n*) located in the spatial resolution is related to the position of both sample point (D) and optical fiber (2πR_f_). Therefore, the total crack width *w* is given by:

(10) $$w=\sum_{i=1}^{n}w_{i}$$

where *w_i_* is the width of each crack located in the spatial resolution and *n* is the total number of cracks located in the fiber whose length is equal to the spatial resolution. Therefore, the crack width can be calculated by the fiber strain of BOTDA.

(11) $$w_{i}=\frac{w}{n}=\frac{\varepsilon \cdot \Delta z}{n}$$

In Equation (11), the width of each crack (*w_i_*) could be obtained. Two assumptions should be mentioned: (1) the number of cracks (*n*) is a known parameter; it can be obtained by logical analysis in the following sections; (2) the width of each crack *w_i_* is an average value.

#### 2.2.4. Optimal Design of Sensors to Monitor the Process of Reinforced Concrete Corrosion

According to the principles discussed above, several key points should be considered in the design of an optimal sensor:

(1) The embedding of an optical fiber into the concrete should cause the least adverse effect on the concrete; therefore, the optical fiber should be as slim as possible. In this paper, a 900-μm tight-buffered optical fiber and 125-μm bare Bragg grating were selected for BOTDA and FBG, respectively.
(2) As bare Bragg grating fiber is extraordinary brittle, effective methods need to be implemented to protect the grating. In this paper, the bare Bragg grating is pasted on the surface with tight-buffered fibers and covered by epoxy resin for protection.
(3) Temperature compensation should be implemented to obtain precise results. Based on Equations (1) and (5), the measured strain is impacted by temperature in both BOTDA and FBG sensing technologies. In this paper, temperature compensation was achieved by setting up compensation samples cast with the same materials, embedded with the same sensors and placed in the same environment as the test samples. However, the compensation samples lacked a steel bar, so that no corrosion-induced concrete expansion or cracking occurred.

### 2.3. Experiment Design

Reinforced concrete corrosion in natural environments is a long-term process, typically occurring over the course of decades. Therefore, electrical accelerated corrosion tests are commonly employed to study reinforced concrete corrosion. However, in these tests, the corrosion products permeate and contaminate the concrete surface, such that it is impossible to identify the initial crack or to measure the crack width. To avoid this issue, we conducted a simulation test based on water-pressure loading. The main purposes of the accelerated corrosion test and water-pressure test were as follows:

(1) Electrical accelerated corrosion test: distributed optical fibers and bare Bragg grating fibers were embedded into samples simultaneously in order to monitor concrete expansion by BOTDA and to identify cracks by FBG.
(2) Water-pressure loading simulation test: distributed optical fibers alone were embedded into samples to establish the relationship between optical fiber strain and crack width.

#### 2.3.1. Water-Pressure Loading Simulation Test

**Figure 3:**
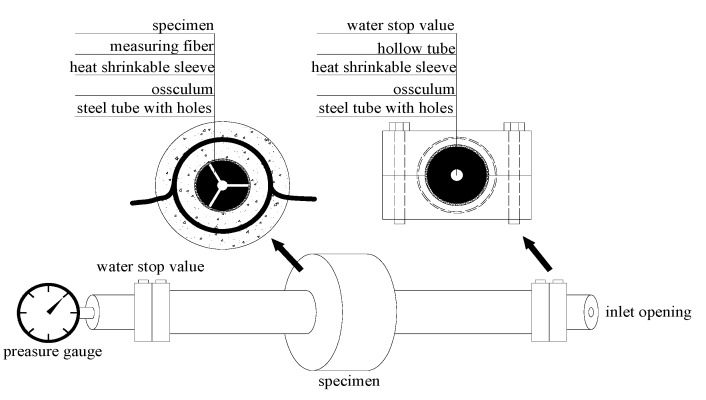
Schematic of the water-pressure simulation test [15] (with permission from Chinese Journal of Sensors and Actuators).

The samples used in the water-pressure loading simulation test were circular rings with an external diameter of 85 mm and an internal diameter of 20 mm. After being wound with 2.0 m of optical fiber, the diameter of the optical fiber ring was 75 mm. A schematic of the water-pressure loading simulation test is shown in Figure 3.

**Figure 4:**
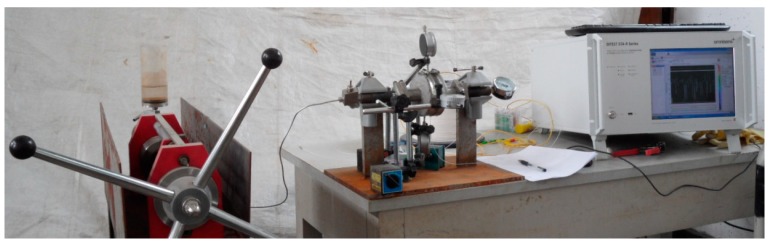
Layout of the water-pressure loading simulation test [15] (with permission from Chinese Journal of Sensors and Actuators).

Water pressure was applied to the internal hole of the circular ring to simulate corrosion-induced expansion force. The expansion force was produced by pouring water into a hollow tube. An inlet opening was installed at one end of the tube, and a pressure gauge was installed at the other end of the tube. In the middle section of the tube, three osculums were arranged around the tube. The water outflow from the three osculums was sealed up by a heat-shrinkable sleeve and a water stop valve. The section of heat-shrinkable sleeve located inside the concrete tube was free, while the other sections were restrained by the steel tube. Thus, the free section could produce an expansion force on the surface of the concrete sample. The layout of the water-pressure loading simulation test is shown in Figure 4.

**Figure 5:**
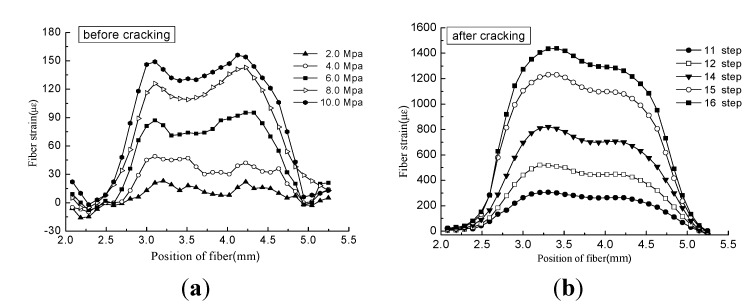
(**a**) Strain of optical fiber before cracking; (**b**) strain of optical fiber after cracking [15] (with permission from Chinese Journal of Sensors and Actuators).

A DITEST STA-R BOTDA (product of OMNISENS Switzerland) was used to monitor the strain of the distributed optical fiber. The spatial resolution was set as 500 mm, and the sampling point distance was set as 100 mm. BOTDA recorded the Brillouin frequency shift automatically during the experiment. Strain was calculated by Equation (1). Previous experiments revealed that cracking would begin once the pressure gauge reading reached 6.0 Mpa–7.0 MPa. This might result in brittle failure if no restraint measure were applied on the surface of the concrete sample. BOTDA cannot record the strain in such a short time; hence, a fastener was installed on the surface of the concrete sample to control the crack propagation. The simulation test occurred via a two-stage process: (1) the surface of the concrete sample remained free; the loading step was set as 1.0 Mpa and was then increased to 6.0 Mpa; (2) a fastener was fixed on the surface; the load was gradually increased until an initial crack was observed, after which the fastener was released step by step. The strain of the optical fiber is shown in Figure 5.

#### 2.3.2. Electrical Accelerated Corrosion Test

**Figure 6:**
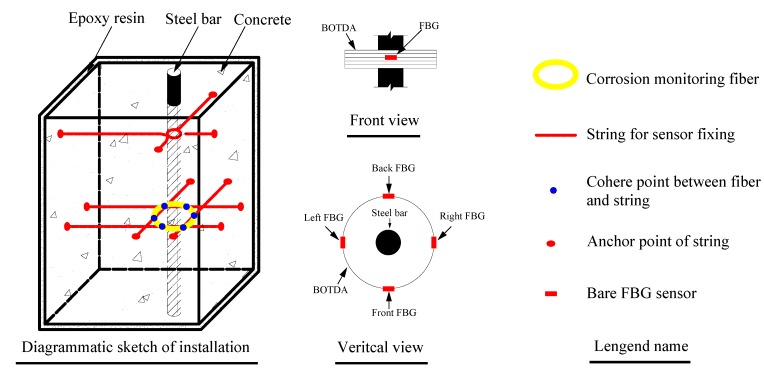
Sample dimensions and optical sensor arrangement. FBG, fiber Bragg grating.

The concrete sample employed in the electrical accelerated test was a cubic specimen with dimensions of 150 mm × 150 mm × 150 mm. The specification of the steel bar was HRB335 with a 20-mm diameter. The detailed configuration of the concrete sample is shown in Figure 6.

The concrete cover was set as 40 mm. As wood formwork was applied to cast the cubic specimen. A hole with a 21-mm diameter was set at the bottom board of the wood formwork. Eleven holes with a 2-mm diameter were drilled at the other four surfaces of wood formwork, which acted as the anchor point for strings. In order to install the steel bar precisely, one end was inserted into the hole on the bottom board, while the other end was fastened by strings. Another network knitted by four strings provided an installation platform for corrosion monitoring fibers. The fibers were made into a loop 75 mm in diameter firstly, then placed into the network at the correct position and, finally, bonded to the string.

**Figure 7:**
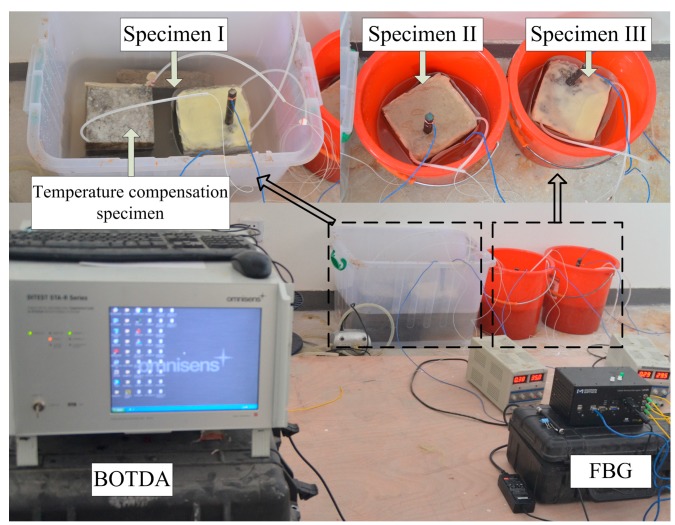
Layout of the electrical accelerated corrosion experiment.

In order to mimic actual conditions as closely as possible, four surfaces of the specimen were painted with epoxy resin. The other two surfaces adjacent to the steel bars were not painted, so that chloride ions could transfer into the concrete sample freely. Three specimens were used in the parallel electrical accelerated corrosion test. Only the distributed optical fiber sensor was embedded in the concrete cover of Specimen I and Specimen III, while the distributed optical fiber sensor and bare Bragg grating were embedded in Specimen II simultaneously. The embedding method for the sensors has been described in Section 2.2. The parameters for the electrical accelerated corrosion test are set as follows: specimen NaCl concentration, 5%; density of direct current, 4000 μA/cm^2^. The layout of the experiment is shown in Figure 7.

A DITEST STA-R BOTDA was used to monitor the strain of the distributed optical fiber. The parameters used were the same as that of the water-pressure loading simulation experiment. A MOI (company named Micron Optics) 125 static FBG was used to monitor the wavelength variation of the bare Bragg grating. A temperature compensation specimen without reinforced steel bar was placed in the same environment as Specimens I, II and III. No steel bar embedded means no expansion and cracking would occur in the temperature compensation specimen. Distributed optical fiber and bare Bragg grating were embedded the same way as Specimen II in the temperature compensation specimen. Thus, the Brillouin frequency shift and Bragg wavelength shift recorded by BOTDA and FBG were only related to the temperature change.

**Figure 8:**
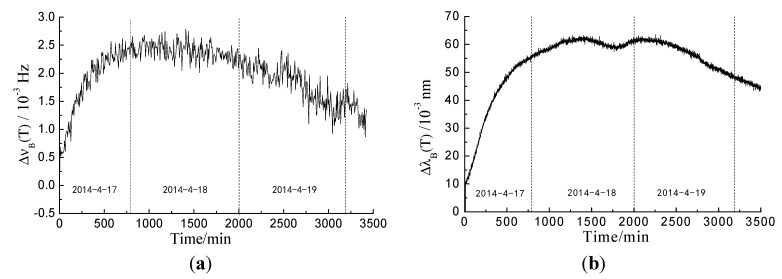
(**a**) Brillouin frequency shift due to temperature of BOTDA; (**b**) wavelength shift due to temperature of FBG.

**Figure 9:**
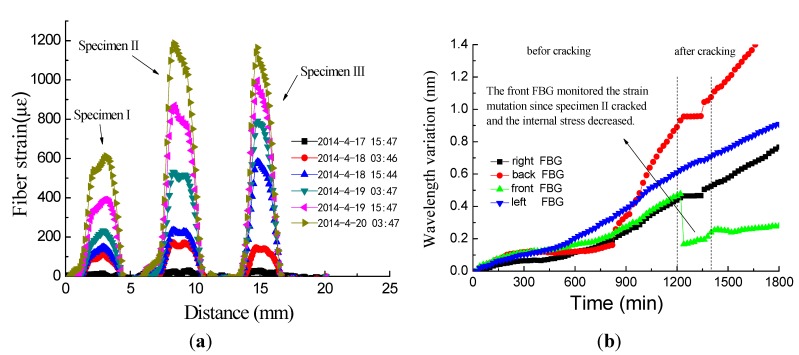
(**a**) Data from BOTDA sensors; (**b**) data from FGB sensors in Specimen II.

In 8a, b, the temperature variation of BOTDA and FBG shares the same tendency. Since the specimens were immersed in water, the temperature variation did not have the same regularity with the change of environmental temperature. The shift values of distributed optical fiber and wavelength are used to acquire temperature compensation according to Equations (1) and (5). The strain of the distributed optical fiber and wavelength variation by considering temperature compensation are shown in Figure 9.

In Figure 9a, the three strain areas represent Specimens I, II and III, respectively. We observed a general trend of increasing strain over time. The data indicate that the strain values were uniform, especially for Specimen I, whose strain values were significantly lower than those of Specimens II and III. Reinforcement corrosion in concrete is a complicated process. Thus, strain would vary among each specimen. The main reasons for the variations are as follows: (1) since the quality of epoxy resin painted on the bottom surface of Specimen I was poorer than the other two specimens, this would cause the corrosion products to leak out and reduce the expansion force indirectly; (2) the corrosion degree varied along the steel bar. Thus, the strain of each specimen was distributed non-uniformly along the length. However, only one monitoring ring was embedded into concrete for each specimen. In Figure 9b, the four strain curves represent the FBG embedded in four different directions around the steel bar in one specimen. As with the data from BOTDA, the wavelength variation increased with time. However, the degree of variation was different for the four curves, as an apparent decline of FBG is observed at the front. A detailed analysis is carried out in the following sections.

## 3. Results and Discussion

### 3.1. Monitoring Concrete Expansion Using BOTDA

**Figure 10:**
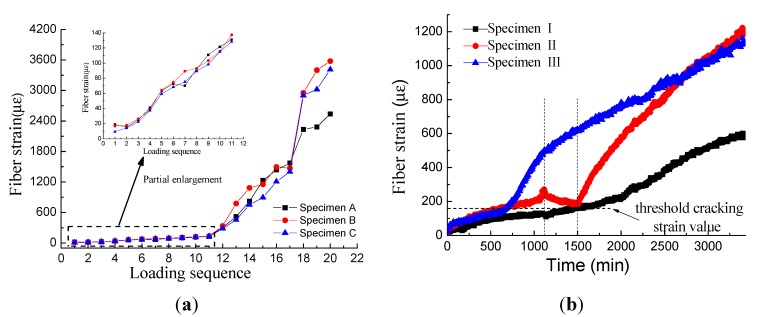
(**a**) Water-pressure test result; (**b**) accelerated corrosion test result.

A water-pressure test and accelerated corrosion test were performed to monitor concrete expansion (Figure 10a, b, respectively).

Figure 10a,b show that the water-pressure test and accelerated corrosion test share the similar curve shape. In Figure 10a, strain increased linearly before the 11th loading step, indicating that the concrete deformation was in the elastic stage. Then, the slope of the strain curve increased remarkably, representing that the concrete cover was in the cracking stage. Three specimens (marked as Specimens A, B and C) with the same concrete mixture were loaded in the water-pressure test. Data fluctuated during the expansion period and the cracking period, respectively. The fluctuation range during the expansion period (the first step to the 11th step) is normally ±20 με, which is acceptable for the BOTDA technique. However, the variation among the three specimens became remarkable during the cracking period. As mentioned in Section 2.3.1, a fastener was fixed on the surface of the concrete to avoid brittle fracture. Since the crack width was controlled by releasing the fastener manually, it is exceedingly difficult to release uniformly. In terms of data, the fiber strain of three specimens after the 11th step was uniform. The water-pressure test proved that the BOTDA technique achieved high precision during the concrete expansion period. The critical value of concrete cracking strain is meaningful for concrete durability evaluation. Additionally, it is feasible to obtain the critical value through the water-pressure test.

From Figure 10a, the average strain of three specimens at the 11th step was 132 με, which was smaller than the critical cracking strain of concrete because of the fastener. Water-pressure tests were also carried out on six specimens without fasteners. The results showed that the threshold value for evaluating cracking was considered as 160 με. However, the value of critical cracking strain depends on the steel bar diameter, the concrete cover depth and the concrete mixture. Thus, the calibration test should be operated firstly to obtain the critical cracking strain of the concrete. In this paper, the parameters of the accelerated corrosion test were set the same as those of the water-pressure test. Thus 160 με in the water-pressure test would act as a reference for the accelerated corrosion test.

However, some differences can be observed between Figure 10a,b. First, the threshold value yielded by the accelerated corrosion test was larger (approximate 180 με), as the corrosion products of the accelerated test permeated more slowly into the pores. Second, the strain curve of the accelerated corrosion test was much more complicated than that of the water-pressure test, especially for Sample II, where the strain decreased from 1000 min to 1500 min. The decrease in strain can be attributed to the fact that no direct current was applied during this period, allowing the corrosion products to permeate the pores without the formation of new corrosion products.

The results above provide a method to evaluate corrosion expansion. The water-pressure test can determine a threshold value that may be used to evaluate the degree of expansion and to predict when cracking will occur.

### 3.2. Identification of Cracking Points by FBG

Before cracks extended to the surface of concrete, only a part of the circumference of the steel bar corrodes. After cracks are induced by corrosion penetrating the concrete cover, steel corrosion has spread across the entire circumference of the rebar [21]. Additionally, some key parameters used for estimating corroded expansion force, such as elastic modulus, are inconstant [22]. The acquisition of time-varying parameters is the key to evaluating concrete durability. However, those parameters, like the rust expansion rate and inconstant elastic modulus, are not only related to the time, but also to the inner crack. The whole corrosion process could be detected by embedding optical fiber hoops of different diameters into the concrete. However, a fiber 900 μm in diameter is too thick and will damage the integrity of concrete. In this paper, the inner cracking process was recorded by the FBG sensor, since the bare brag grating of 125 μm in diameter is slim enough. In practical structural health monitoring, the corrosion cracks are always observed on the bottom surface of the member. As mentioned above, the inner cracking process is useful for achieving time-varying parameters, and recognizing inner cracks is helpful for concrete durability evaluation.

Because the BOTDA method for evaluating cracking is limited in its spatial resolution, it is difficult to identify the positions of corrosion cracks. To overcome this limitation, bare Bragg grating was embedded with distributed optical fibers to monitor strain variations at four different positions. The embedding method and experimental process are discussed in Section 2.2.

The results are shown in Figure 9b. Data from the four bare Bragg gratings were quite different. For the sensor in the back, the wavelength increased over time (with the exception of the time period between 1200 and 1380 min), and the degree of change was larger than that of the other three sensors. For the sensors located at the front, left and right, data variation trends were fairly similar prior to 1200 min, as corrosion products permeated into the concrete pores and produced sustained concrete expansion. However, between 1200 and 1380 min, the wavelength decreased or remained constant. The decrease in wavelength was especially significant for the front sensor. Cracks appeared at the front surface during this stage, which may be the main cause of this large decrease in wavelength. The decreased amplitude of the wavelength is related to the distance between the sensor and cracks, and this phenomenon would be the criterion for recognizing cracking positions.

The results above provide a method to recognize cracking positions. Bare Bragg grating embedded in the concrete around the steel bar allowed us to identify cracking positions by analyzing the degree of wavelength decrease.

### 3.3. Monitoring Crack Width Using BOTDA

**Figure 11:**
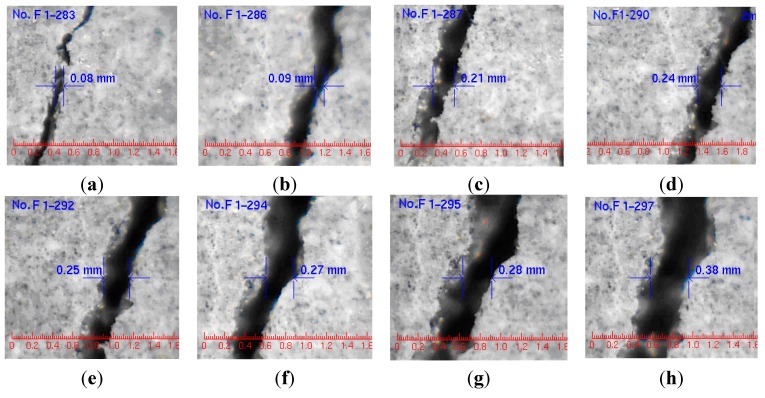
Width variation of one crack in Specimen A: (**a**) 12 steps; (**b**) 13 steps; (**c**) 14 steps; (**d**) 15 steps; (**e**) 16 steps; (**f**) 17 steps; (**g**) 18 steps; (**h**) 19 steps [15] (with permission from Chinese Journal of Sensors and Actuators).

In addition to the monitoring of concrete expansion and identification of points of initial cracking, the monitoring of crack width is also important for the evaluation of concrete durability. Since specimens from an accelerated corrosion test are contaminated by corrosion products, it is impossible to determine the crack width manually in these samples. Thus, a water-pressure experiment was carried out to monitor crack width (Figure 11).

**Figure 12:**
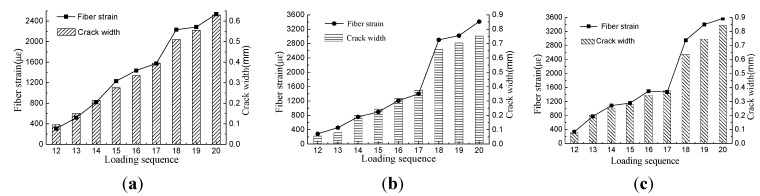
Comparison of optical fiber strain and crack width. (**a**) Data of Specimen A; (**b**) data of Specimen B; (**c**) data of Specimen C.

Crack observation instruments were able to record the crack width precisely. The initial cracking width was 0.08 mm and increased with increasing water pressure. The strain of the distributed optical fiber is shown in Figure 10. Optical fiber strain and crack width of Specimens A, B and C are compared in Figure 12a–c, respectively.

**Figure 13:**
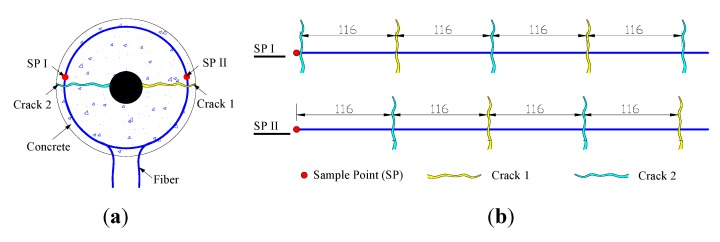
Illustration for crack detection by BOTDA: (**a**) sectional view; (**b**) unfolded view.

Figure 12 shows a similar increasing trend between optical fiber strain and crack width for the three specimens. For Specimens B and C, the releasing process was quicker than Specimen A. The fiber strain and crack width of Specimens B and C both increased suddenly at the 18th step, and their agreement degree was lower after the 18th step. Since the cracking process was controlled by releasing the fastener manually, it was impossible to keep the same loading process for the three specimens. The linear relationship between optical fiber strain and crack width was obvious for Specimen A during the whole loading progress and was obvious for Specimens B and C during the first 17 steps, as well. The fitting equations are as follows:

(12) $$\text{For Specimen A: }w=242\varepsilon ,R^{2}=0.988$$

(13) $$\text{For Specimen B: }w=263\varepsilon ,R^{2}=0.966$$

(14) $$\text{For Specimen C: }w=243\varepsilon ,R^{2}=0.980$$

where *w* is crack width (the total widths of all cracks), *ε* is optical fiber strain and *R* is the correlation coefficient. Therefore, estimating crack width by fiber strain of BOTDA is feasible. It should be noted that the crack width is the summation of all crack widths around the specimen, shown as Figure 13a:

In the water-pressure test, two cracks appeared simultaneously, which exhibited an approximately symmetrical distribution. Thus, the relationship between average crack width and fiber strain is as follows:

(15) $$\text{For Specimen A: }w_{1}=121.0\varepsilon ,R^{2}=0.988$$

(16) $$\text{For Specimen B: }w_{1}=131.5\varepsilon ,R^{2}=0.966$$

(17) $$\text{For Specimen C: }w_{1}=121.5\varepsilon ,R^{2}=0.980$$

Shown as Figure 13b, five cracks cross the fiber at the sample Point I, while four cracks cross the fiber at the sample Point II. This phenomenon can explain the non-uniform distribution of strain in Figure 5b. In other words, the number of cracks located in the fiber whose length is equal to the spatial resolution is different for each sample point. The strain in Figure 10 is the maximal value from Figure 5b. In this case, cracks crossed through the fiber five times, so the parameter *n* in Equation 11 equals five. The theoretical relationship between average width and fiber strain is as Equation 18:

(18) $$w_{1}=\frac{\varepsilon \cdot \Delta z}{n}=\frac{\varepsilon \cdot 500}{5}=100\varepsilon$$

By comparing Equation 17 with Equation 15, the maximal error of the crack is 31.5. This means that if the fiber strain were 2000 με, a 0.063-mm estimated error of the crack width would occur. Additionally, this error is acceptable.

## 4. Conclusions

In this paper, we present a novel sensor for reinforced concrete corrosion based on BOTDA and FBG. This sensor can monitor the entire corrosion process, including concrete expansion, and can detect the initial position and crack width. Based on the data from the two types of experiments carried out to evaluate this novel sensor, we conclude that:

(1) Optical fibers are characteristically anti-corrosive and stable, rendering them suitable as candidates for sensors in corrosive environments. We present the theoretical principles underlying sensor function based on elastic mechanics and geometric analysis, and we show that the theoretical and experimental results matched reasonably.
(2) Corrosion-induced concrete expansion can be monitored by BOTDA. A water-pressure test can be employed to obtain the threshold value for evaluating cracking, and this value may be used to evaluate the degree of expansion and to predict when cracking occurs.
(3) Corrosion-induced initial cracking can be recognized by FBG. Using a bare Bragg grating embedded in concrete around the steel bar, cracking positions could be determined by analyzing the degree of wavelength decrease.
(4) Corrosion-induced crack width can be detected by BOTDA. The coefficient between optical fiber strain and crack width can be obtained using theoretical equations and experimental parameters. Corrosion crack width can be estimated by analyzing the optical fiber strain from BOTDA.
(5) In this paper, the familiar variation trend of fiber strain from BOTDA and wavelength from FBG are observed before cracking. This indicated that FBG alone may be able to monitor the whole corrosion process, thus the monitoring costs would become reasonable, because the equipment for BOTDA is extremely expensive. Furthermore, some calibration experiments should be carried out to establish the relationship between concrete strain and wavelength.

It should be noted that the threshold value for concrete cracking depends on the concrete mixture, while the cracking width coefficient depends on the test parameters of BOTDA. Thus, a calibration experiment should be carried out firstly before applying this novel sensor to a specific project. The experiments reported in this paper were conducted indoors; it would therefore be necessary to perform these experiments again on-site to study the performance of the sensor in a different setting.
